# Implementation of a system-wide health promotion intervention to reduce early mortality in high risk adults with serious mental illness and obesity

**DOI:** 10.1186/1748-5908-10-S1-A15

**Published:** 2015-08-20

**Authors:** Stephen Bartels, Mary Brunette, Kelly Aschbrenner, Gail Daumit

**Affiliations:** 1 Geisel School of Medicine at Dartmouth, Dartmouth Centers for Health and Aging, Lebanon, NH, 03766, USA; 2 Johns Hopkins School of Medicine, Johns Hopkins University, Baltimore, MD, USA

## Overview

Persons with serious mental illness (SMI) experience a high rate of medical comorbidity, resulting in reduced life expectancy of 7-30 years less than that of the general population largely due to cardiovascular disease. High rates of obesity and smoking are major factors contributing to early mortality, associated with sedentary behavior, poor diet, and prescription by physicians of high-weight-gain antipsychotic medications. This panel will describe a multicomponent approach to addressing one of the nation's leading health disparities through a rigorous statewide implementation effort aimed at transforming mental health services by integrating health promotion and obesity prevention through organizational change, prescriber behavior change, and patient engagement in service design and implementation. The first presentation on organizational change describes how the results of an NIMH-funded RCT of the In SHAPE health promotion intervention were applied to a statewide implementation study including process outcomes of a learning collaborative, system level (organizational change) outcomes, and patient-level outcomes. The second presentation on prescriber behavior change will describe outcomes of a statewide academic detailing/audit and feedback intervention to decrease prescribing of high weight gain propensity antipsychotic agents for individuals with SMI. The third presentation on patient-peer engagement describes the results of a pilot study designed by patients, providers and academic researchers with the goal of increasing uptake and reach of the professionally-led In SHAPE health coach fitness model by phasing out one-on-one fitness coach training sessions with individual and group-based peer support, motivational text messages, and physical activity sensors for monitoring and feedback. The discussion will consider how these findings may inform future systems change, national efforts, and implementation research to improve the health and longevity of high-risk adults with SMI and cardiovascular risk factors.

## Organizational change and participant outcomes in a statewide implementation of a health promotion intervention for serious mental illness and obesity

### Objective

To describe organizational change and patient-level outcomes of a statewide implementation of health promotion addressing obesity in persons with serious mental illness (SMI) in public mental health settings.

### Method

We evaluated the statewide implementation of an evidence-based health promotion program (In SHAPE) through a naturalistic study involving: (1) assessments of patient-level outcomes for 120 mental health consumers and (2) assessments of organizational change through in-depth interviews with senior leaders, program directors, and staff at participating community mental health centers (CMHCs). The stepped implementation created a naturally occurring "intervention group" consisting of one urban and one rural CMHC and a "usual care" comparison group with the same geographic composition. Participants were assessed at baseline, 6-, and 12-month follow-up. Organizational change assessments were conducted during the implementation phase at each of the four sites at baseline, 6-, 12-, and 24-month follow-up. Implementation was assessed based on ratings on the General Organizational Index adapted for In SHAPE consisting of 11 organizational change domains.

### Findings

Major barriers to implementing health promotion programming at CMHCs were financing, competing priorities, and integrating health promotion into team operations. Facilitators of implementation were leadership buy-in, organizational culture prioritizing wellness, staff interest, and high consumer demand. Mean GOI score (range 1-5) increased from baseline (1.9) to 2-year follow-up (3.7) with higher organizational change associated with high program uptake. Implementation vs. waitlist organizations were associated with significantly greater weight loss, increased fitness, increased vigorous exercise and physical activity, and dietary change.

### Impact

Organizational change supporting integrated health promotion may reduce cardiovascular risk among obese adults with serious mental illness. Based on these promising results we have been recently funded to compare the effectiveness of a virtual IHI Learning Collaborative vs. targeted technical assistance in implementing health promotion (In SHAPE) in 48 mental health organizations across the nation.

## Academic detailing and audit and feedback to decrease prescribing of high weight gain antipsychotic medications

### Objective

To evaluate changes in prescribing behavior associated with an academic detailing/audit and feedback intervention occurring in a statewide health promotion program implementation.

### Method

We assessed whether three in-person sessions of group academic detailing with audit and feedback could improve prescribing across a state mental health system. Using Medicaid claims for antipsychotic medications, we conducted regression discontinuity analyses assessing changes in the proportion of people getting antipsychotics with low cardiometabolic risk, high cardiometabolic risk, or in combination with other antipsychotics among all Medicaid recipients getting antipsychotics, comparing the year before and the year after the intervention. Additional regression analyses were conducted over a five-year period 2009-13 to assess longer term changes.

### Findings

Discontinuity analyses demonstrated a significant decrease in the proportion of people in community mental health center treatment obtaining high-risk antipsychotic agents following the initiation of the prescriber intervention.

### Impact

Our data suggest that academic detailing focused on evidence-based prescribing with audit and feedback on prescribing practices can shift the pattern of antipsychotic utilization in public mental health settings.

## Peer support and technology to enhance sustainability and reach of community-based health promotion for obese persons with serious mental illness

### Objective

To partner with key stakeholder groups to adapt and pilot test a novel health promotion intervention enhanced with peer support and technology delivered in a public mental health center.

### Method

Academic researchers partnered with peer support specialists and mental health and fitness providers to design and implement an adapted version of the professionally-led In SHAPE health coach model that included phasing out one-on-one coached fitness training sessions with individual and group-based peer support and exercise, motivational text messages, and physical activity sensors for monitoring and feedback. Ten participants were recruited and assessed at baseline and post-intervention using a mixed-methods framework. Feasibility was evaluated through qualitative interviews with key stakeholders in addition to a preliminary exploration of descriptive health outcomes.

### Findings

The peer support and technology-enhanced model was desirable, feasible, and associated with possible improvements in exercise, physical activity, healthy eating, and readiness to change health behaviors.

### Impact

Incorporating "wellness peers" and mHealth technology into health promotion programs for persons with serious mental illness holds promise for enhancing program sustainability and reach. This pilot data furthers our understanding of how professionally-driven models of health promotion can be adapted and delivered by "peers" in public mental health settings to potentially extend the reach valuable health promotion resources to a high-risk patient population.

## Funding

NIMH R01 MH078052 "Health Promotion and Fitness for Younger and Older Adults" with SMI (PI Bartels) NIMH R01 MH089811 "Statewide Intervention to Reduce Early Mortality in Persons with Mental Illness" (PI Bartels).

